# MiR-195 suppresses non-small cell lung cancer by targeting CHEK1

**DOI:** 10.18632/oncotarget.3255

**Published:** 2015-03-25

**Authors:** Ben Liu, Jinli Qu, Fangxiu Xu, Yan Guo, Yu Wang, Herbert Yu, Biyun Qian

**Affiliations:** ^1^ Department of Epidemiology and Biostatistics, Key Laboratory of Breast Cancer Prevention and Therapy, Ministry of Education, Key Laboratory of Cancer Prevention and Therapy, Tianjin, National Clinical Research Center of Cancer, Tianjin Medical University Cancer Institute and Hospital, Tianjin 300060, China; ^2^ Cancer Epidemiology Program, University of Hawaii Cancer Center, Honolulu, HI 96813, USA; ^3^ Hongqiao International Institute of Medicine, Shanghai Tongren Hospital and Faculty of Public Health, Shanghai Jiao Tong University School of Medicine, Shanghai 200025, China

**Keywords:** non-small cell lung cancer, miR-195, CHEK1, prognosis, cell cycle

## Abstract

MiR-195 suppresses tumor growth and is associated with better survival outcomes in several malignancies including non-small cell lung cancer (NSCLC). Our previous study showed high miR-195 plasma levels associated with favorable overall survival of non-smoking women with lung adenocarcinoma. To further elucidate role of miR-195 in NSCLC, we conducted *in vitro* experiment as well as clinical studies in a cohort of 299 NSCLC samples. We demonstrated that miR-195 expression was lower in tumor tissues and was associated with poor survival outcome. Overexpression of miR-195 suppressed tumor cell growth, migration and invasion. We discovered that CHEK1 was a direct target of miR-195, which decreased CHEK1 expression in lung cancer cells. High expression of CHEK1 in lung tumors was associated with poor overall survival. Our results suggest that miR-195 suppresses NSCLC and predicts lung cancer prognosis.

## INTRODUCTION

Lung cancer is the most common malignancy and leading cause of cancer death in the world [1]. Non-small cell lung cancer (NSCLC) is a major class of lung cancer in which adenocarcinoma and squamous cell carcinoma account for the majority of tumor histology. Despite extensive research and significant improvement in early detection and treatment options, the disease is still difficult to treat and many patients develop recurrent diseases after surgery. Less than 15% of the patients with advanced disease can survive 5 years after diagnosis [2, 3]. For these reasons, many recent studies focused on finding new prognosis biomarker and pivotal molecular associated with development and metastases of lung cancer [4, 5].

MicroRNAs (miRNAs) are small (18 to 24 nucleotides in length), single-stranded, endogenous non-coding RNAs that regulate gene expression post-transcriptionally. Mammalian miRNAs are generally encoded in the introns of pre-messenger RNAs (pre-mRNAs) or 3′ untranslated regions of messenger RNAs (mRNAs). They suppress gene expression by binding to the complementary regions of mRNAs, which either blocks translation or facilitates mRNA degradation through the RNA-induced silencing complex [6]. Recent reports have summarized that aberrant expression of miRNAs has been associated with carcinogenesis and tumor progression [7, 8].

*MiR-195* is a member of the *miR-15/16* family, which consists of a group of miRNAs (*miR-195*, *miR-15a*, *miR-15b*, *miR-16-1* and *miR-16-2*) that share a similar seed sequence [9]. The sequence of mature *miR-195* is conserved across mammalian species [10]. Previous studies have shown aberrant *miR-195* expression in multiple cancer sites, including breast cancer [11], hepatocellular carcinoma [12], colorectal cancer [13, 14], gastric cancer [15] and NSCLC [16]. Like other members of the *miR-15/16* family, *miR-195* has been reported to have different, sometime conflicting, effects on cell growth and apoptosis in cancer. The role of *miR-195* in NSCLC, however, remains unclear. In one of our previous studies, we investigated levels and clinical implications of several miRNAs in the circulation of non-smoking women with lung adenocarcinoma [17], and found high plasma levels of *miR-195* associated with better overall survival. These observations led us to further investigate the role of *miR-195* in NSCLC. In this investigation, we first confirmed that *miR-195* expression was low in NSCLC compared to adjacent non-tumor tissues and low expression was associated with poor prognosis. We then showed in our *in vitro* experiments that increasing *miR-195* expression in lung cancer cells suppressed cell proliferation, migration and invasion. We also identified a target of *miR-195*, *CHEK1*, and demonstrated that *miR-195* down-regulated its expression and delayed cell cycle progression in lung cancer cells.

## RESULTS

### Patient characteristics

Clinical features of NSCLC patients in this study are summarized in Table 1. Patient median follow-up time was 35.2 months (range between 0.6 and 82.8 months), and mean age at diagnosis was 61 years old, ranging from 34 to 83 years. Patient median overall survivals were significantly different by age at diagnosis (shorter in older patients), histological type (shorter in squamous cell carcinoma) and disease stage (shorter in advanced stage) (Table 1). Of these patients, tumor expression of *miR-195* and CHEK1 were analyzed in 85 and 276, respectively.

**Table 1:** Characteristics and survival outcome of patients

Variables	Number (%)	Death	MST (M)^e^	Log-rank *P*	HR (95% CI)	HR^f^(95% CI)
Age at diagnosis (*n* = 299)						
< 60	138 (46.15%)	58	65.47	**0.011**	1.00	1.00
≥ 60	161 (53.85%)	86	35.47		**1.54 (1.10–2.15)**	**1.62 (1.11–2.36)**
Gender (*n* = 299)						
Male	178 (59.53%)	93	52.17	0.164	1.00	1.00
Female	121 (40.47%)	51	52.70		0.78 (0.56–1.11)	0.89 (0.53–1.50)
Smoking history (*n* = 299)						
No	119 (39.80%)	52	52.70	0.270	1.00	1.00
Yes	180 (60.20%)	92	52.17		1.21 (0.86–1.71)	0.85 (0.48–1.49)
Histology subtype (*n* = 299)						
SCC^a^	142 (47.49%)	74	53.50	**0.034**	1.00	1.00
ADC^b^	121 (40.47%)	48	72.00		**0.68 (0.47–0.97)**	**0.52 (0.33–0.83)**
Others^c^	36 (12.04%)	0	–	–	–	–
TNM stage (*n* = 298)^d^						
I + II	188 (63.09%)	68	44.70	< **0.001**	1.00	1.00
III + IV	110 (36.91%)	75	24.47		**2.58 (1.85–3.60)**	**2.74 (1.89–3.97)**
*miR-195* (*n* = 85)						
Low expression	39 (45.88%)	26	16.26	**0.025**	1.00	1.00
High expression	46 (54.12%)	23	52.70		**0.53 (0.30–0.93)**	**0.44 (0.24–0.81)**
CHEK1 (*n* = 276)						
Low expression	155 (56.16%)	70/85	65.46	**0.037**	1.00	1.00
High expression	121 (43.84%)	63/58	35.86		**1.44 (1.02–2.02)**	**1.42 (1.00–2.02)**

a SCC: squamous cell carcinoma.

b ADC: adenocarcinoma.

c No statistics are computed because of no death among these cases

d Numbers do not equal to the total number due to missing data.

e MST (M): medium survival time (months).

f Adjusted for gender, age at diagnosis, smoking history, family history, histological type, and TNM stage.

### *MiR-195* expression in NSCLC

**Figure 1:**
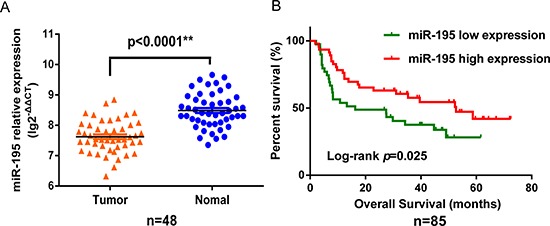
MiR-195 expression in NSCLC and association with survival *MiR-195* was down-regulated in NSCLC and low expression of *miR-195* was associated with poor survival. **(A)** *MiR-195* levels in 48 pairs of tumor and adjacent non-tumor tissues, measured by RT–qPCR, referenced to *RNU6B*. **(B)** Kaplan–Meier overall survival curves by high and low *miR-195* expression in 85 NSCLC patients.

In analysis of 48 paired tumor and adjacent non-tumor tissue samples, we found *miR-195* expression was significantly higher in tumor tissues than in adjacent non-tumor tissues (*p* < 0.0001; Figure 1A). Survival analysis showed that *miR-195* expression in 85 tumor samples was significantly associated with the overall survival of NSCLC patients. Patients with high *miR-195* expression had better overall survival compared to those with low expression (*p* = 0.025; Figure 1B), and the hazard ratio (HR) was 0.53 (*p* = 0.025), a nearly 50% reduction in risk for death. This association remained significant after adjusting for TNM stage, histology, smoking history, and family history of cancer (HR, 0.44; 95%CI, 0.24–0.81) (Table 1).

### Effects of *miR-195* expression on cell proliferation

**Figure 2:**
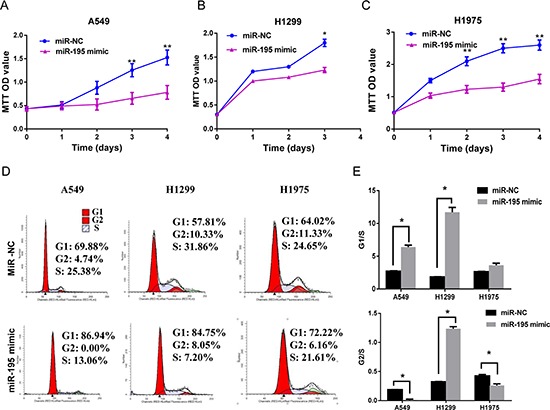
MiR-195 expression, cell proliferation and cell-cycle arrest in lung cancer cells Increasing *miR-195* expression in lung cancer cell lines reduced cell proliferation and induced cell-cycle arrest. Cell lines A549 **(A)**, H1299 **(B)** and H1975 **(C)** were transfected with *miR-195* or *miR-NC*, and cell proliferation was measured by the MTT assay. Cell-cycle analysis was performed by flow cytometer to determine the impact of *miR-195* or *miR-NC* on cell-cycle progression. The representative flow cytometry patterns in 3 cell lines were shown in **(D)** and the G1/S and G2/S ratios in each cell line were shown in **(E)** (*n* = 3).

After transfecting lung cancer cells (A549, H1299 and H1975) with *miR-195* mimic or scrambled miRNA control (*miR-NC*), we quantified cell numbers with the MTT assay. The results showed that compared to the miRNA control, the number of viable cells was clearly reduced overtime in all 3 cell lines transfected with *miR-195* mimic (*p* < 0.05) (Figure 2A, 2B and 2C), suggesting that cell proliferation was significantly suppressed by *miR-195*. Furthermore, using flow cytometry to assess cell-cycle status, we found that cells transfected with *miR-195* had increased cell numbers in the G1 and G2 phases, but reduced numbers in the S phase (Figure 2D). Analysis of the G1/S and G2/S ratios suggested that *miR-195*-related cell-cycle arrest occurred both in the G1 and G2 checkpoints for A549 and H1299, but only in G2 for H1975 (Figure 2E).

### Effects of *miR-195* expression on cell migration and invasion

**Figure 3:**
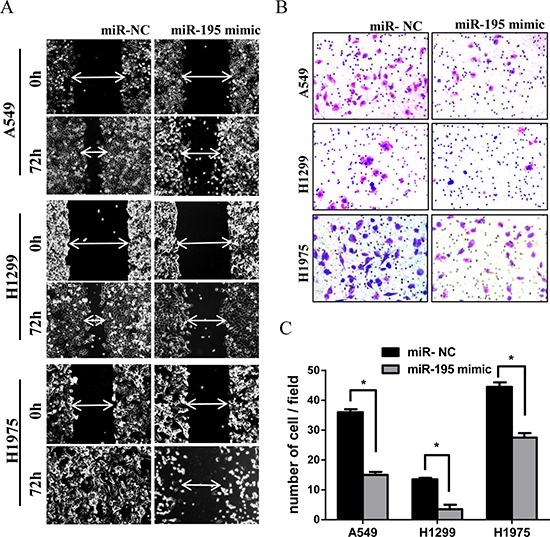
MiR-195 expression and lung cancer cell migration and invasion Increasing *miR-195* expression in lung cancer cell lines suppressed cell migration and inhibited cell invasion. **(A)** In the wound healing assay, A549, H1299 and H1975 cell lines transfected with *miR-195* or *miR-NC* were seeded in the 6-well dishes, and a scratched wound was applied at 24 hours post-transfection. Cell migration to the scratched wound was measured at 0 and 72 hours and compared between cells transfected with *miR-195* and *miR-NC*. **(B)** In the trans-well invasion assay, A549, H1299 and H1975 cell lines transfected with *miR-195* or *miR-NC* were seeded to the mitrigel coated invasion upper chambers at 24 hours post-transfection and allowed to invade toward the other side of chambers for 18–22 hours. The invading cells underneath the chambers were stained and counted. Representative images under microscopy from different experimental conditions were taken from each cell line. **(C)** All histograms show invading cell numbers per microscopic field from three independent experiments. *p < 0.05.

To assess the effect of *miR-195* on cell migration and invasion, we performed the wound healing and trans-well assays on cancer cells transfected with *miR-195* mimic or with *miR-NC*. The wound healing assay showed that *miR-195* expression appeared to inhibit cell migration in H1975, A549 and H1299 cells (Figure 3A). In the trans-well assay, increased *miR-195* expression could reduce cell invasion in all three cancer cell lines (Figure 3B and 3C).

### *CHEK1*, a *miR-195* target in NSCLC cells

**Figure 4:**
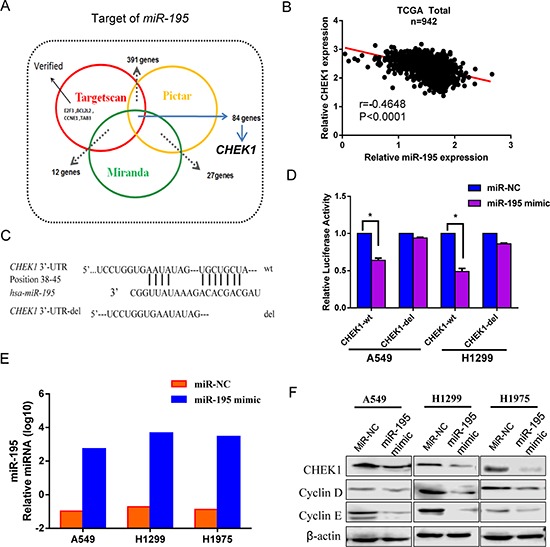
MiR-195 expression and effects on CHEK1 as a target of miR-195 *MiR-195* bound directly to the *CHEK1* 3′-UTRs and down-regulated its expression along with other proteins. **(A)** A Venn diagram shows 3 software which predict miRNA targets and identified 84 candidate genes which may interact with *miR-195*. **(B)** A significant inverse correlation was found in NSCLC between *miR-195* and CHEK1 expression in TCGA. **(C)** A putative *miR-195*-binding site exists in the 3′-UTR of the *CHEK1* mRNA, and 7-nucleotide deletion were generated in the binding site. **(D)** Transfection of *miR-195* inhibited the firefly luciferase activity of the pMIR-REPORT-3′-UTR-CHEK1 (wt), but such inhibition was absent for the reporter which had deletion in the *miR-195*-binding site (del). *MiR-NC* was used as a negative control in all the experiments. The impact of *miR-195* on CHEK1 expression was normalized and compared to those of negative miRNA (*n* = 3, *p* < 0.001). **(E)** The expression of *miR-195* determined by RT-qPCR in three NSCLC cell lines was significantly increased following *miR-195* transfection. **(F)** The protein level of CHEK1 was decreased in three NSCLC cell lines when transfected with *miR-195* with beta-actin as a loading control. Two positive control Cyclin D1 and Cyclin E were detected as known targets of *miR-195*.

Using three miRNA databases, we identified a putative *miR-195*-binding site located in the 3′-UTR of *CHEK1* mRNA (Figure 4A). To further validate the association between *miR-195* and *CHEK1*, we analyzed the TCGA dataset and the result showed that the inverse correlation between *miR-195* and CHEK1 was significant (*r* = –0.46, *p* < 0.0001) in NSCLC samples (Figure 4B). To confirm if *miR-195* directly binds to this location in *CHEK1*, we cloned a full-length *CHEK1* 3′-UTR and inserted it into a luciferase reporter vector, downstream from the firefly luciferase gene. As for control, we made a mutant *CHEK1* 3′-UTR clone which had a 7-nucleotide deletion in the *miR-195* binding site (Figure 4C), and the mutant clone was inserted into the same vector. Both vectors were transfected into lung cancer cells A549 and H1299 together with *miR-195* or *miR-NC*. Our experiments showed that *miR-195* significantly suppressed the luciferase activity in the *CHEK1* wild type clone compared to *miR-NC*, but not in the mutant one (Figure 4D), suggesting that *miR-195* directly binds to the 3′-UTR of *CHEK1* mRNA. The transfection efficiency was examined with RT-qPCR, and our evaluation confirmed that *miR-195* expression was increased substantially in the cell lines transfected with *miR-195* mimic, but not in those with *miR-NC* (Figure 4E).

To further confirm the effect of *miR-195* on *CHEK1*, we analyzed protein levels of CHEK1 by western blot in A549, H1299 and H1975 cells transfected with *miR-195* or *miR-NC*. As known targets of *miR-195*, levels of cyclin D1 and cyclin E proteins were also measured in the cell lines using the western blot. Our protein analyses showed that all these proteins, CHEK1, cyclin D1 and cyclin E, were declined in the *miR-195* transfected cell lines compared to the negative controls (Figure 4F), suggesting that *miR-195* not only directly bind to *CHEK1*, lowering mRNA expression, but also down-regulate its protein level along with other molecules involved in cell cycle regulation.

### CHEK1 expression and NSCLC survival

**Figure 5:**
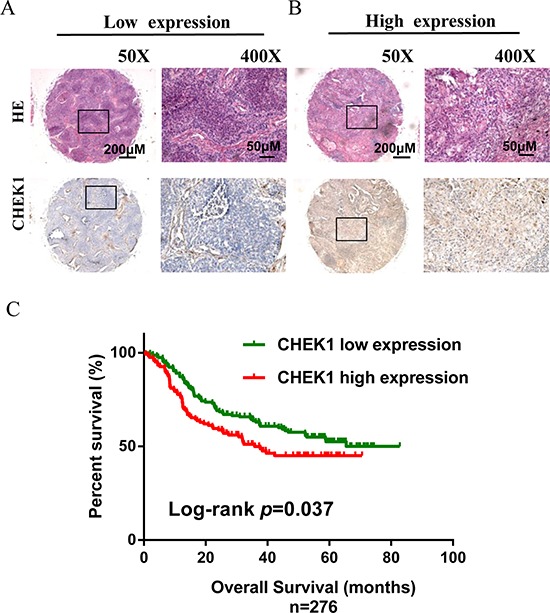
CHEK1 protein expression measured by immunehistochemical staining in tissue microarray and its association with lung cancer survival **(A, B)** Examples of NSCLC tissue samples stained with H&E or specific CHEK1 antibody under low (50×) and high (400×) power microscope; low CHEK1 expression was shown in panel A and high was in panel B (Scale bars represent 200 μm and 50 μm, respectively). **(C)** Kaplan–Meier overall survival curves according to low and high CHEK1 protein expression in 276 cases. Green line represents low protein expression, and red line represents high protein expression.

To assess if CHEK1 expression in NSCLC was associated with patient survival, we measured CHEK1 expression in 276 tumor samples with immunohistochemical staining (IHC). Results of representative tumor samples stained with high and low CHEK1 expression are shown in Figure 5A and 5B. CHEK1 expression was slightly higher in tumor than in adjacent non-tumor tissues, but the difference was not statistically significant (*p* = 0.832) (Data not shown). Compared to low expression, however, high CHEK1 expression was significantly associated with poor overall survival (*p* = 0.037) (Figure 5C). Cox regression analysis confirmed that CHEK1 was associated with survival after adjusting for confounding variables (HR, 1.42; 95% CI, 1.00–2.02) (Table 1). No statistically significant associations were found between CHEK1 expression and clinicopathological features of NSCLC (Supplementary Table 1).

## DISCUSSION

Our tissue analysis showed that *miR-195* expression was lower in tumor than in adjacent non-tumor tissues and low expression in tumor tissues was associated with unfavorable overall survival of NSCLC patients. These observations were in agreement with our previous finding of high circulating *miR-195* being associated with favorable prognosis of non-smoking women with NSCLC [17]. Our *in vitro* experiments suggest that *miR-195* plays an obviously suppressive role in tumor cell proliferation, migration and invasion. Our bioinformatic analysis and cell culture experiments further indicate that the tumor suppressing effects of *miR-195* were mediated in part through its down-regulation of *CHEK1* mRNA, a cell cycle modulator. We also found that high levels of CHEK1 protein in tumor tissues were associated with poor NSCLC survival. To our knowledge, this is the first study to demonstrate the post-transcriptional regulation of CHEK1 by *miR-195* in NSCLC, and their associations with patient survival.

Although two studies reported that *miR-195* expression was increased in chronic lymphocytic leukemia and breast cancer [18, 19], most previous studies showed decreased *miR-195* expression in several types of cancer, including gastric cancer, breast cancer, colon cancer, hepatocellular carcinoma, adrenocortical carcinoma and squamous cell carcinoma of the tongue [12, 20–24]. More recently, Luo et al [11] reported low expression of *miR-195* in breast cancer specimens compared to adjacent non-tumor tissues, suggesting that the miRNA functions as a tumor suppressor. Another recent study examined miRNA signature in bladder cancer (BC) using deep sequencing. The study demonstrated that *miR-195* was down-regulated in BC and could inhibit BC cell proliferation, migration and invasion [25]. These findings were consistent with the observations we made in our clinical studies and cell culture experiments.

Our *in vitro* experiments showed that increased *miR-195* expression could inhibit cell proliferation, suppress cell migration and invasion, and possibly induce cell cycle arrest in the G1 or G2 phases. Similar observations have been reported in a number of cancer cells including NSCLC [16, 26, 27]. The expression of CHEK1 can be regulated by *miR-195* in cardiomyocytes and human epidermoid carcinoma cell line reported by two groups respectively [28, 29]. However, no studies has yet examined that CHEK1 is a potential target of *miR-195* in NSCLC. Our investigation not only demonstrated that *miR-195* interacted with *CHEK1* mRNA and suppressed its protein expression in cancer cells, but also showed that CHEK1 expression was associated with patient survival and the direction of the association was consistent with the action of *miR-195*. *CHEK1* encodes a serine/threonine kinase (also known as Chk1) which is a central component of the DNA damage response. CHEK1 regulates cell cycle checkpoints, and coordinates cellular activities involving DNA repair and cell cycle arrest [30]. In our study, we found that high expression of CHEK1 was associated with poor prognosis of NSCLC; similar associations were observed in ovarian cancer [31, 32].

CHEK1 has been considered a potential target for cancer therapy. CHEK1 inhibitors have been tested as therapeutic agents for several types of cancer including lung cancer, and the test results show that the inhibitors may affect the sensitivity of radiotherapy and chemotherapy [33–36]. Studies also suggest that *miR-195* and CHEK1-related signal pathways have been involved in the sensitivity of chemotherapy to breast cancer [37], laryngeal cancer [38] and colon cancer [13]. So far, CHEK1 inhibitors have shown promise in several preclinical models, and have been tested in a number of ongoing or completed phase I and II clinical trials [39–42]. Taken together, we speculate that reducing CHEK1 activity and increasing *miR-195* expression work in concert suppressing tumor growth.

In addition to *CHEK1*, *miR-195* also regulates cell cycle by targeting other messenger RNAs. Recently, *miR-195* is reported to suppress cell cycle and tumorigenesis through the control of molecules involved in the G1/S phase transition [43, 44], including cyclin D1, CDK4, CDK6, and E2F3. In our experiments, we also found that cyclin D1 and cyclin E were down-regulated by *miR-195*, which was consistent with the observations made by other investigators [11, 44–46]. Earlier experiments also suggest that *miR-195* has additional targets, such as MYB [16], BCL-2 [47], IKKa and TAB3 [26].

To evaluate the validity of our findings, we downloaded microRNA and mRNA expression data from the Cancer Genome Atlas (TCGA). Our analysis showed down-regulation of *miR-195* (Supplementary Figure 1A) and up-regulation of *CHEK1* (Supplementary Figure 1B) in tumor samples in comparison to adjacent tissues. Furthermore, high *CHEK1* expression was associated with poor survival of NSCLC patients (*p* = 0.031) (Supplementary Figure 1D). No association, however, was found between *miR-195* and survival (*p* = 0.208) (Supplementary Figure 1C) in TCGA.

In conclusion, we found that NSCLC had lower *miR-195* expression in tumor than in adjacent tissues and low expression was associated with poor overall survival. This finding was consistent with our previous observation of *miR-195* in plasma. Our cell culture experiments of lung cancer cells showed that increased *miR-195* expression could suppress cell proliferation, migration and invasion. We also found that *miR-195* was able to bind to *CHEK1* mRNA down-regulating its expression. Low CHEK1 protein was associated with favorable survival of patients with NSCLC. Collectively, high *miR-195* and low CHEK1 work synergistically suppressing tumor growth and improving the survival outcome of NSCLC patients.

This investigation has led to several novel observations. First, to our best knowledge, this is the first report showing that *miR-195* suppressed NSCLC through, at least partially, down-regulating the expression of *CHEK1*, a newly discovered target in lung cancer. Furthermore, it is novel to know that *miR-195* and CHEK1 can be independent prognostic factors in NSCLC. Thus, of particular interest, the *miR-195*/CHEK1 axis might represent a new molecular target for NSCLC treatment.

## MATERIALS AND METHODS

### Patients and tumor samples

Patients in the study were recruited from the Tianjin Medical University Cancer Hospital (TMUCH) between May, 2006 and July, 2011. During the time, we recruited 299 newly diagnosed patients who had histologically confirmed non-small cell lung cancer (NSCLC). Each patient provided tissue samples for the study, and the specimens were collected during tumor resection. The tissue samples were histologically confirmed to be tumor or non-tumor tissues, and were stored at −80°C until analysis. All patients enrolled in the study were followed from surgery to August 28, 2013 through scheduled office visits and regular telephone contacts. Information on histology, tumor size, disease stage, lymph node involvement, distant metastasis, and treatments was extracted from patient medical records and pathology reports. Demographic features, tobacco use and family history of cancer were collected using a structured questionnaire. The study was approved by the medical ethics committee at TMUCH.

### RNA extraction and analysis

Total RNA was extracted from the collected fresh-frozen tissue specimens using the standard Trizol method; miRNA expression was measured with the microRNA assay (Applied Biosystems Inc, US). The expression analysis is described briefly as follows. First, 25 ng of total RNA were reverse-transcribed to cDNA using the TaqMan^®^ MicroRNA Reverse Transcription Kit (Applied Biosystems Inc., US). The reverse transcription (RT) was completed sequentially under the following conditions: incubation at 16°C for 30 minute, 42°C for 30 minute and 85°C for 5 minute. After RT, quantitative polymerase chain reaction (qPCR) was performed in the 7900 HT-Fast real-time PCR system (Applied Biosystems Inc., US) following the protocol of denaturing at 95°C for 10 minute, and 40 cycles of denaturing at 95°C for 15 second and annealing and elongation at 60°C for 1 minute. The qPCR results were analyzed with the SDS Relative Quantification Software version 2.1 (Applied BioSystems Inc., US). Small RNA RNU6 was utilized as an endogenous control to normalize the quantity of cDNA used for analysis of *miR-195* expression. All samples were analyzed in triplicate, and the measurement was repeated if the coefficient of variation was greater than 5%. The expression level of *miR-195* was calculated based on the formula 2^−△△Ct^, where ∆Ct = Ct _(*miR-195*)_–Ct_(RNU6)_.

### Cell culture and transfection

Three lung cancer cell lines, A549, H1299 and H1975, were selected for our cell culture experiments. These cell lines were maintained in RPMI-1640 (GIBCO, US), supplemented with 10% (V/V) fetal bovine serum (FBS) (GIBCO, US), and the culture media were changed every 3 days. For *in vitro* experiments, cells were seeded at 3 × 10^5^ per well in the 6-well plates, 5 × 10^4^ per well in the 24-well plates, and 2 × 10^3^ per well in the 96-well plates, and allowed to attach for at least 24 hours. To assess the effects of *miR-195* on cell activities, 50 nM *miR-195* mimic or scrambled *miRNA* control (*miR*-NC) (Shanghai GenePharma, China) were transfected to the cells using the Lipofectamine RNAiMAX (Invitrogen, US) according to the manufacturer's instruction. At 6 hours post-transfection, culture media were replaced with those containing 10% FBS. *CHEK1* 3′-UTR were cloned into the pMIR-REPORT Luciferase plasmids (Promega, US) which were transfected into the lung cancer cells using the DharmaFECT Transfection Reagent (Thermo, US).

### Luciferase reporter assays

A549 and H1299 cells maintained in the 24-well plates were transfected with 0.4 μg of the pMIR-REPORT Luciferase plasmids and miRNA using the Lipofectamine 2000 (Invitrogen, US). The relative luminescences were measured 24 hours post-transfection using the Dual Luciferase Reporter Assay Kit (Promega, US).

### MTT assay

Cell proliferation was measured by the MTT assay (Nanjing KeyGEN Biotech, China). After being seeded in the 96-well plates for 24 hours, cells were transfected with *miR-195* mimics or miRNA controls. At 24, 48, 72 and 96 hours of post-transfection, cells were gently washed with PBS, and 20 μl MTT (5 mg/ml) were added in the cell culture. After 4 hours of incubation, the media were discarded, and 150 μl DMSO was added in each well to dissolve the precipitates. The absorbance of the resulting solution was measured at 590 nm wavelength with a microplate reader (Bioteck, US). Each experimental condition was carried out in 6 replicates, and repeated 3 times.

### Wound healing assay

Cell migration was measured using the wound healing assay in the 6-well plates. A fine line was scraped with a 10 μl tip in each well after cultured cells became fully confluent. After scratch, cells were continuously cultured in the media with 3% FBS for 72 hours. Microscopic pictures of the cultures were taken at 0, 24, 48 and 72 hours. The experiments were performed 3 times independently.

### Trans-well assay

To analyze cell migration, cultured cells were re-seeded at a concentration of 5 × 10^4^ onto the upper chamber of an 8 mm pore size insert (BD, US). The inserts were placed in the wells of a 24-well plate, and the cells were cultured in serum free medium. For invasion analysis, 1 × 10^5^ cells were added onto the upper chamber of matrigel-coated (BD, US) 8 mm pore size inserts placed in the wells of a 24-well plate, and the cells were cultured in the media containing 10% FBS. After 24 hours of incubation, cells migrated through the membrane or invaded through the matrigel into the lower chamber of the insert were fixed with 75% ethanol for 30 min and stained with crystal violet. After taking photograph from five random visual fields, cells were counted in each photo. These experiments were repeated 3 times.

### Cell-cycle analysis

Cultured cells were seeded onto the 6-well plates at a density of 1 × 10^6^ cells per well and incubated overnight. After 48 hours of transfection, cells were collected and washed with PBS. Cell pellets were fixed in 70% cold ethanol overnight at −20°C. The fixed cells were washed in PBS and resuspended in the Cell Cycle Reagent (Millipore, US) at 5 × 10^5^ cells/ml. The cells were incubated in the dark for 15 minutes at room temperature. The cell solutions were analyzed by a flow cytometer, guava easyCyte™ (Millipore, US), to determine cell populations at different cell cycle phases. The DNA contents of the stained cells were analyzed using the Modfit LT software (Verity Software House, US).

### Tissue microarray and immunohistochemical staining

CHEK1 protein expression in tumor samples was measured with immunohistochemical staining. Tissue microarrays (TMA) were constructed from the archived formalin-fixed paraffin-embedded tissue blocks using the “TMA Builder” (Beecher Instruments, USA). A total of 15 slides were developed which contained both tumor and adjacent non-tumor tissues from 276 patients with NSCLC. Each tissue sample contributed at least two 0.6 mm cores, and each core represented either tumor or adjacent non-tumor tissue, determined by the study pathologists who examined the corresponding sections stained with hematoxylin & eosin. TMA slides were first stained with a CHEK1 antibody. This primary antibody was then interacted by a secondary antibody conjugated with horseradish peroxidase from the EnVision Detection Systems (DAKO, Denmark). After formation of the antigen-antibody-antibody complex, a substrate of the peroxidase, diaminobenzidine, was added as chromogen. The tissue staining was done according to the manufacturer's instructions. All the TMA slides were counterstained with hematoxylin. TMA staining results were evaluated independently by two pathologists, and the samples with different results were re-evaluated until a consensus was reached. The staining conditions for TMA were optimized when both positive and negative cells were present in the same tissue sample. Signals were considered positive when the reaction products were localized in the expected cellular component. The tissue staining results were scored based on signal distribution (distribution score) and intensity (intensity score). The distribution score includes 0 (0–5%), 1 (6–25%), 2 (26–50%), 3 (51–75%) and 4 (76–100%), which indicates the percentage of positive cells in all the tumor cells present in a sample. The signal intensity consists of 0 (no signal), 1 (weak), 2 (moderate), or 3 (strong). The final staining score was the product of distribution and intensity scores.

### Western blot analysis

Western blot was performed for detection of specific proteins in our cell culture experiments. The protocol started with uploading 30 μg proteins from the whole cell lysate in each sample onto a 10% PAGE gel. After electrophoresis and gel transferring, the membrane was blocked with 5% non-fat milk in 1xTris-buffered saline (pH 7.4) containing 0.05% Tween-20, and then probed with primary antibodies at concentrations of 1:1000 for β-actin (Santa Cruz Biotechnology, US), 1:2000 for CHEK1 and Cyclin E and 1:10000 Cyclin D1 (both from Epitomics, US). Secondary antibodies were added at concentrations of 1:10,000 to 1:20,000. The detected proteins were visualized using the Visualizer Western Blot Detection Kit (Millipore, US).

### TCGA data analysis

To evaluate the validity of our findings, we downloaded microRNA and mRNA expression data and their corresponding clinical information from the Cancer Genome Atlas (TCGA) (https://tcga-data.nci.nih.gov/tcga/) in August, 2014. The microRNA expression data generated from the Illumina Genome Analyzer and HiSeq 2000 contained 956 NSCLC tumors and 91 normal lung tissue samples. The mRNA expression data generated from the Illumina HiSeq 2000 involved 977 NSCLC tumors and 108 normal lung tissue samples. The expression data were processed with quantile normalization using the preprocessCore in the R/Bioconductor package [48]. Samples and corresponding clinical data were linked by tumor barcodes. Survival analyses were performed on NSCLC tumor samples that had follow-up information using the SAS software. Pairwise comparisons were evaluated with *t*-test.

### Statistical analysis

The study results were analyzed using the statistical software SAS (version 9.1.3, SAS) and SPSS (version 16.0, SPSS Inc.). RNA expression was calculated as expression index (EI) based on the formula 2^−△△Ct^. The EI was analyzed as a continuous variable. Independent two-sample Student's *t*-test was used to compare the differences in *miR-195* expression by clinicopathological features of NSCLC. Kaplan–Meier survival curves were compared between patients with high and low expression of biomarkers using the log-rank test. Associations between molecular markers and NSCLC survival were also examined with the Cox proportional hazards regression model at both univariate and multivariate levels. In the Cox regression analysis, *miR-195* and CHEK1 were categorized into high and low groups using their medians as cutoff. Differences were considered statistically significant when a *p* value was less than 0.05. All *p* values reported were two-side.

## SUPPLEMENTARY FIGURE


