# Supplementary material for: MiR-195 suppresses non-small cell lung cancer by targeting CHEK1
Source: Oncotarget. 2015 Mar 25;6(11):9445–56. doi: 10.18632/oncotarget.3255 (PMC4496229; doi:10.18632/oncotarget.3255)
Supplement: Supplementary file 1 [file oncotarget-06-9445-s001.pdf]

SUPPLEMENTARY FIGURE AND TABLE

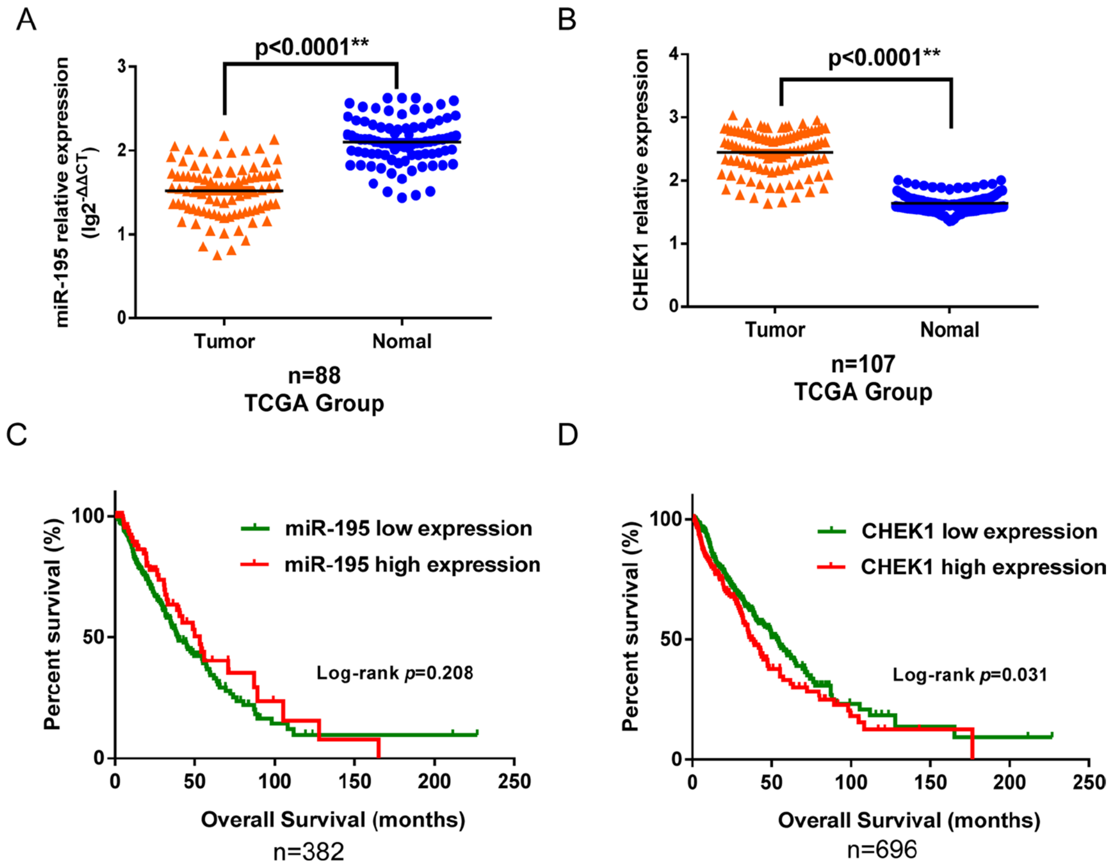

Supplementary Figure 1: Expression of *miR-195* and *CHEK1* in TCGA data and their associations with one another and NSCLC survival. **(A)** *MiR-195* expression was lower in NSCLC tumor than in adjacent non-tumor tissues in TCGA. **(B)** *CHEK1* expression was higher in NSCLC tumor than in adjacent non-tumor tissues in TCGA. **(C)** Kaplan–Meier overall survival curves in 382 NSCLC patients from TCGA were not significantly different ($p = 0.208$) between high and low *miR-195* expression. **(D)** Kaplan–Meier overall survival curves in 696 NSCLC patients from TCGA were significantly different ($p = 0.031$) between high and low *CHEK1* expression.

Supplementary Table 1: Associations of *miR-195* and *CHEK1* expression and clinicopathological features of NSCLC patients

Variables	*miR-195* expression (*n* = 85)				*CHEK1* expression (*n* = 276)			
	Total	Low	High	*P* value	Total	Low	High	*P* value
Age at diagnosis								
≤ 60	39	19	20	0.629	137	79	58	0.617
> 60	46	20	26		139	76	63	
Gender								
Male	42	22	20	0.235	169	97	72	0.603
Female	43	17	26		107	58	49	
Family history of cancer								
No	67	28	39	0.144	224	125	99	0.661
Yes	18	11	7		49	29	20	
Smoking history								
No	45	20	25	0.778	103	61	42	0.429
Yes	40	19	21		173	94	79	
Histology subtype								
SCC[a]	36	15	21	0.621	139	77	62	0.768
ADC[b]	41	21	20		106	62	44	
others	8	3	5		31	16	15	
TNM stage								
I–II	48	23	25	0.569	178	102	76	0.606
III–IV	36	15	21		98	53	45	
Tumor infiltration								
T1T2	74	33	41	0.537	201	112	89	0.81
T3T4	11	6	5		75	43	32	
Lymph node metastasis								
N0	40	21	19	0.113	157	94	63	0.201
N1–N3	43	16	27		117	61	56	
Distant metastasis								
M0	78	35	43	0.533	261	145	116	0.399
M1	7	4	3		15	10	5	
Tumor size								
≤ 3 cm	26	12	14	0.973	88	49	39	0.942
> 3 cm	59	27	32		187	105	82	

[a]SCC: Squamous cell carcinoma
[b]ADC: Adenocarcinoma
